# Cooling down for going up: Could selective ‘brain chilling’ mitigate high‐altitude illness?

**DOI:** 10.1113/EP093165

**Published:** 2025-12-04

**Authors:** Adnan Haq, Damian M. Bailey

**Affiliations:** ^1^ Neurovascular Research Laboratory, Faculty of Life Sciences and Education University of South Wales Pontypridd UK; ^2^ Bexorg, Inc. New Haven Connecticut USA

The rising popularity of mountaineering and high‐altitude (HA) expeditioning comes at a price: many people will get sick. The ever‐present risk of HA illness, ranging from benign albeit debilitating forms of acute mountain sickness (AMS) to potentially life‐threatening syndromes of high‐altitude cerebral oedema (HACE) and pulmonary oedema (HAPE), have the collective potential to impact the tens of thousands of enthusiasts each year when they ascend above 2500 m above sea‐level. Mountaineering and Netflix fans alike may be familiar with the documentary ‘14 Peaks: Nothing Is Impossible’, which captures some of the challenges faced during the ‘Project Possible’ climbs – the main star, Nirmal ‘Nims’ Purja, developed HACE, but thankfully managed to survived.

Pharmacological management of these HA syndromes focuses on prevention, symptomatic treatment and emergency management (Bärtsch & Swenson, 2013). Acetazolamide, a carbonic anhydrase inhibitor, remains the mainstay for AMS prophylaxis and treatment, enhancing ventilation through metabolic acidosis due to enhanced renal bicarbonate excretion (Leaf & Goldfarb, 2007). This compensates for the respiratory alkalosis caused by hypoxia‐induced hyperventilation and increases carotid chemoreceptor sensitivity. Ventilation and systemic oxygenation are consequently elevated, as well as cerebral blood flow (CBF) and substrate delivery, serving to mitigate the debilitating symptoms of AMS (Fan et al., 2012). Typical dosing is 125–250 mg twice daily, initiated before ascent and continued during exposure. For moderate to severe AMS or established HACE, dexamethasone (4 mg every 6 h) is effective due to its anti‐inflammatory and anti‐oedematous effects on the brain (Luks & Hackett, 2022). In contrast, HAPE, characterised by exaggerated hypoxic pulmonary vasoconstriction (Bailey et al., 2010), responds to pulmonary vasodilators such as nifedipine (20 mg slow‐release every 8 h) (Bärtsch et al., 1991) and phosphodiesterase inhibitors including sildenafil and tadalafil (Ghofrani et al., 2004) that collectively reduce pulmonary artery pressure and improve oxygenation. Despite the pharmacological options, descent and supplemental oxygen remain the most definitive and practical interventions. Analgesics and antiemetics offer symptomatic relief, while portable hyperbaric chambers provide temporary support when descent is not immediately feasible. However, altitude medication is not without limitations, with common adverse effects including insomnia, nausea, headache, paraesthesia, hypotension, kidney stones and gastrointestinal discomfort.

Given the popularity of mountaineering expeditions, the heterogeneous nature of HA illness aetiology, their rapid onset following hypoxic exposure and the importance of prioritising prevention over cure, it would be prudent to explore additional, complementary countermeasures. Brain cooling (i.e., therapeutic hypothermia) has demonstrated promise in neuroprotection and clinical settings – for example, in mitigating hypoxic brain injury in neonates (Shankaran et al., 2005) and traumatic brain injury in sports‐related concussion (Al‐Husseini et al., 2023; Walter et al., 2023), albeit with unconfirmed efficacy based on large scale randomised controlled trials. However, its application in managing (i.e., preventing or delaying) HA illness remains less clear.

Brain cooling exerts its neuroprotective benefits subsequent to mitochondrial bioenergetic suppression characterised by a reduction in the cerebral metabolic rates of oxygen ($\mathrm{CM}R_{O_{2}}$) and glucose (CMR_Glu_) (initially due to tissue temperature‐induced reductions on enzymatic activity and cerebral O_2_ extraction) as well as a reduction in CBF (Erecinska et al., 2003; Laptook et al., 2001). The premise is that by cooling the brain down and reducing metabolic activity, mitochondrial dysfunction will be mitigated, thereby preventing excessive formation of free radicals. Free radicals are thermodynamically poised to cause membrane destabilisation and structural damage when in physiological excess, notwithstanding neutralising the primary vasodilator, nitric oxide (Cobley et al., 2018), resulting in what is collectively termed oxidative‐inflammatory‐nitrosative stress (OXINOS) (Bailey et al., 2013). Reductions in $\mathrm{CM}R_{O_{2}}$ and CMR_Glu_ will be closely coupled with CBF in the context of brain hypothermia due to the impact of metabolic autoregulation and local respiratory alkalosis (occurring independently of changes in cerebral O_2_ extraction) – i.e. reduced CO_2_ production will liberate fewer H^+^ ions and increase pH triggering L‐type Ca^2^⁺ channel activation, increasing intracellular Ca^2^⁺ in cerebral artery vascular smooth muscle cells to effect cerebral vasoconstriction (Dabertrand et al., 2012; Kontos et al., 1977).

Most neuroprotective effects of brain cooling have been observed with profound brain hypothermia – typically <34°C. However, a 1°C reduction in brain temperature confers a 5%–9% reduction in $\mathrm{CM}R_{O_{2}}$ (Erecinska et al., 2003), according to the *Q*_10_ temperature coefficient principle, which stipulates the rate of change of biochemical reactions engendered by a 10°C tissue temperature change (Mundim et al., 2020). Some data suggest a *Q*_10_ value of ∼2.5 in the mammalian brain (Russell‐Buckland & Tachtsidis, 2020), although this remains open to debate (Erecinska et al., 2003). Thus, milder brain cooling (e.g., 35–36°C) may be sufficient to attain a ‘clinically meaningful’ suppression in free radical formation while capitalising on their hormetic, neuroprotective signalling benefits when in physiologically controlled, albeit undefined concentrations (Bailey et al., 2011). Local cerebral and pulmonary OXINOS is a pathogenic risk factor for HA illness (Bailey et al., 2009b, 2009a, 2010), thus it is theorised that mitigating OXINOS via cooling could prove a useful preventative measure. This approach could broaden the range of strategies available for managing HA illness, providing reassurance to expeditioners who may be reluctant or unable to rely on pharmacological interventions.

Other means of brain cooling that exert neuroprotective effects include anti‐inflammation (further mitigating OXINOS), curtailing oedema, apoptosis and astrocytic damage (Deng et al., 2003; González‐Ibarra et al., 2011; Wang et al., 2015). Despite the emergent evidence for therapeutic hypothermia protecting against blood–brain barrier (BBB) disruption (Baumann et al., 2009), it is worth recalling that BBB disruption is not a universal feature in AMS, notwithstanding the limitations of current neuroimaging modalities and sensitivity–specificity of blood‐borne molecular biomarkers (Janigro et al., 2021). There is also evidence to suggest that brain cooling can reduce intracranial pressure (Polderman et al., 2002), yet the role of intracranial hypertension in AMS and HACE has been challenged due to evidence illustrating that altered redox homeostasis may be the unifying mechanism underlying HA pathophysiology (Bailey et al., 2009b, 2009a). Excessive free radical formation can contribute to Na⁺/K⁺‐ATPase pump failure (Biller et al., 2021; Rohn et al., 1996), and endothelial dysfunction subsequent to elevated OXINOS (Bailey et al., 2019). This is also considered the proximal catalyst for pulmonary vasoconstriction, hypertension and corresponding development of HAPE (Bailey et al., 2010; Smith & Schumacker, 2019). Nonetheless, the overall uncertainty in the pathophysiology of AMS and particularly HACE raises challenges in coupling the potential therapeutic hypothermia‐induced neuroprotection with HA disorder progression.

An additional consideration relates to the optimal brain cooling method. Systemic cooling (e.g., deep hypothermic circulatory arrest) has illustrated benefits for hypoxic encephalopathy (Eicher et al., 2005), aortic arch repair (Jubouri et al., 2025) and cerebral outcomes post‐cardiac arrest (Hypothermia after Cardiac Arrest Study Group, 2002) using external cooling devices (e.g., a mattress that delivers cold air throughout a patient's entire body). However, the practical challenges and clinical complications (e.g., hyperglycaemia, shivering, bradycardia and electrolyte abnormalities) associated with whole body hypothermia make selective brain cooling techniques preferable. Intravascular saline and intranasal administration may work, particularly the innovative ‘RhinoChill’ system, which has gained utility in a variety of clinical settings (Islam et al., 2015), demonstrating brain temperature reductions of 1.4°C within an hour in brain‐injured patients with minimal adverse effects (Abou‐Chebl et al., 2011). However, these methods may be deemed overly invasive for the otherwise healthy mountaineer. Surface head/neck cooling has also demonstrated favourable results with regards to reducing brain temperature whilst maintaining core temperature with improved neurological outcomes. For instance, a fluid‐filled cervical cooling collar was recently shown to significantly reduce a brain–core temperature gradient by 30%, as well as improve cerebrovascular reactivity (Lavinio et al., 2024). Additionally, cooling helmet application in head injury and stroke patients induced an average brain temperature reduction of 1.84°C within an hour without adverse effects (Wang et al., 2004). Collectively, these exciting findings highlight the potential value of non‐invasive targeted brain cooling methods for benefiting HA expeditions.

Although targeted brain cooling can reduce brain temperature while maintaining core temperature, the role of the cerebral circulation in mediating brain thermoregulation could limit its efficacy, due to elevated CBF in hypoxia acting as a heat source in an attempt to restore brain temperature (Wang et al., 2014). It would therefore be prudent to consider the timing of brain cooling treatments during HA expeditions to impact the net CBF effect – that is, by initiating cooling early enough, hypoxic cerebral vasodilation could be pre‐empted and reductions in $\mathrm{CM}R_{O_{2}}$ may suppress CBF sufficiently to limit heat delivery to the brain. This would enhance the net cooling effect and help curtail OXINOS before it escalates. It is also emphasised that selective brain cooling should not be applied concurrently with the aforementioned treatment options, notably acetazolamide, due to the potentially conflicting mechanisms of action – i.e. impacts on CBF (Lassen et al., 1987).

Another physiological limitation is that selective brain cooling may not be adequate to lower arterial $P_{CO_{2}}$, and thus CBF, given their close interdependence (Carr et al., 2023). While hyperventilation at altitude reduces arterial $P_{CO_{2}}$, leading to cerebral vasoconstriction, this effect is often overridden by the dominant influence of hypoxia‐induced vasodilation (Rupp et al., 2014). Consequently, global CBF may remain elevated despite hypocapnia (Liu et al., 2017; Subudhi et al., 2010), potentially increasing AMS susceptibility through a flow‐mediated rise in cerebral free radical output for any given arterio‐jugular venous difference (Bailey et al., 2009a) and/or sensitisation of the meninges leading to cephalalgia (Bailey et al., 2009c). Therefore, the assumption that low $P_{CO_{2}}$ necessarily equates to reduced CBF may not hold in all cases, particularly in the context of altitude‐related pathophysiology. This also raises the question of whether $P_{CO_{2}}$ plays a critical role in the local CBF reductions observed with targeted head cooling and underscores the importance of distinguishing between systemic and local mechanisms. Comparing the efficacy of systemic versus selective brain cooling remains challenging due to the numerous variables involved, and there is currently no consensus on the optimal approach. Further discussion of various cooling techniques, including their advantages and limitations, is available elsewhere (Assis et al., 2019; Hong et al., 2022). Table 1 compares the relative merits of selective brain cooling against oxygen supplementation, whilst Figure 1 illustrates the physiological rationale for the potential application of brain cooling in the management of HA disorders.

**FIGURE 1:**
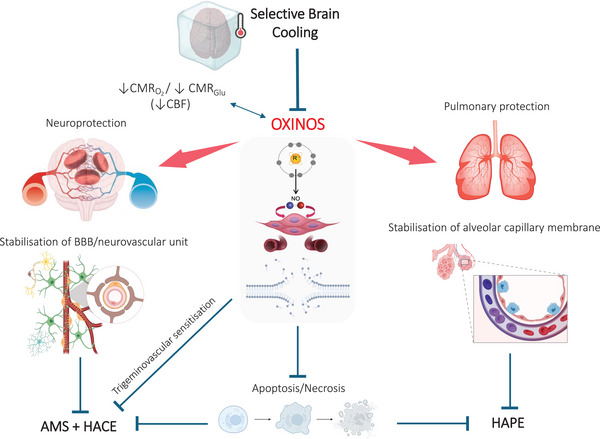
Potential neuroprophylactic benefits of selective brain cooling (targeted therapeutic hypothermia) to mitigate high‐altitude (HA) illness. Selective brain cooling reduces $\mathrm{CM}R_{O_{2}}$/CMR_Glu_ due to the *Q*_10_ principle and corresponding CBF due to metabolic autoregulation, whereby perfusion is matched to metabolic demand and adjusted due to changes in CO_2_ output and local $P_{CO_{2}}$. This curtails mitochondrial induction of potentially harmful free radicals and corresponding oxidative–inflammatory–nitrosative stress (OXINOS), the unifying upstream catalyst for cerebral dysfunction. Attenuated systemic/local OXINOS can also downregulate mitochondrial function and thereby $\mathrm{CM}R_{O_{2}}$ and CMR_Glu_ (bi‐directional pathway) and reduce free radical formation, increase vascular nitric oxide bioavailability and/or mitigate necrotic/apoptotic cellular death. Reduced OXINOS serves to stabilise key components of the neurovascular unit, notably the BBB, astrocytes and/or Na^+^,K^+^‐ATPase channels, thereby curtailing cytotoxic and/or vasogenic oedema and ultimately HACE. OXINOS can also contribute to AMS independent of structural deformation of the neurovascular unit due to activation of trigeminovascular pain pathways, causing headache, its defining symptom. Although unclear, HAPE progression could also be delayed due to the established role of free radicals in triggering hypoxic pulmonary vasoconstriction and pulmonary arterial hypertension. AMS, acute mountain sickness; BBB, blood–brain barrier; CBF, cerebral blood flow; $\mathrm{CM}R_{O_{2}}$, cerebral metabolic rate of oxygen; CMR_Glu_, cerebral metabolic rate of glucose; HACE, high‐altitude cerebral oedema; HAPE, high‐altitude pulmonary oedema.

Targeted technological advances are needed to enhance practical utility. Lightweight, portable cooling systems – such as Peltier‐type helmets capable of selectively cooling brain regions without disturbing core temperature regulation (Tauchi et al., 2018; Wang et al., 2004) – warrant further exploration. Integrating these systems with biosensors to monitor cortical oxygenation, temperature, and OXINOS biomarkers could increase their applicability. Controlled hypoxia trials in environmental chambers, combined with neuroimaging and haematological assessments, would then help clarify mechanistic pathways before field validation in mountaineering settings.

While a more focused effort to elucidate the neuroprotective mechanisms of brain cooling in the context of HA illness appears eminently justified, several pertinent questions and uncertainties remain. First, it is not yet clear whether the magnitude of brain cooling‐induced reductions in systemic/local OXINOS is sufficient to meaningfully influence the progression of AMS or HACE. Second, the optimal method (including application timing) of achieving effective and portable brain cooling in field settings has yet to be determined. Third, the practicalities of asking mountaineers to carry brain cooling equipment on arduous treks may present logistical hurdles – and perhaps raise a few eyebrows. Finally, the potential safety and ethical implications of such interventions require careful scrutiny. Future research should therefore: (1) validate non‐invasive selective brain cooling methods in hypoxia; (2) quantify cerebral metabolic and vascular responses, including free radical generation, during cooling; and (3) assess feasibility, tolerability and potential symptom mitigation in pilot laboratory and field trials. Although proposed primarily as a preventative or symptom‐delaying approach rather than a treatment for established AMS or HACE, the utility of brain cooling remains speculative. Whether brain chill can help mountaineers scale the mental and physical hill remains an open – and icy – question!

**TABLE 1:** Comparison of selective brain cooling methods to oxygen supplementation for the management of high‐altitude illness.

Feature	Selective brain cooling	Oxygen supplementation
**Mechanism of action and evidence**	↓ $\mathrm{CM}R_{O_{2}}$, ↓ OXINOS, ↑ BBB protection Cooling collars, helmets, RhinoChill → ↓ brain temp ↓ CBF/$\mathrm{CM}R_{O_{2}}$ ↑cerebral autoregulation (Abou‐Chebl et al., 2011; Lavinio et al., 2024; Wang et al., 2004)	RCTs report physiological (↑ $S_{pO_{2}}$, ↑ cerebral perfusion, and ↑ cerebral oxygenation) and cognitive improvements (PVT and DSST) (Falla et al., 2024; Kovacs et al., 2021)
**AMS/HACE management**	No published clinical trials to date	Strong clinical support and guideline‐backed (Luks et al., 2023)
**Logistics/portability**	Requires equipment, setup and monitoring Helmets and cooling collars – moderate to high portability – weight typically 2 kg including coolant/battery Rhinochill – low portability	Immediate, widely used portability depends on size of oxygen cylinders/concentrators – weight typically 2–10 kg
**Cost**	Cooling helmets (e.g., Peltier type) – ∼£300–£1000 RhinoChill – ∼£10,000	Cylinders – £200–£300 + refills. Concentrators – £1000–£3000
**Possible adverse effects**	Helmets – ↑ BP and mucosal irritation RhinoChill – ↑ BP, mucosal irritation, and invasive (Hong et al., 2022; Koehn et al., 2012)	May curtail acclimatization (e.g., EPO) Dry nasal passages or mucosal irritation Headaches or mild dizziness (Khalife et al., 2021; Lindvåg Lie et al., 2022; Miyamoto & Nishimura, 2008)

Abbreviations: AMS, acute mountain sickness; BBB, blood–brain barrier; BP, blood pressure; CBF, cerebral blood flow; $\mathrm{CM}R_{O_{2}}$, cerebral metabolic rate of oxygen; DSST, Digit‐Symbol Substitution Test; EPO, erythropoietin; HACE, high‐altitude cerebral oedema; OXINOS, oxidative–inflammatory–nitrosative stress; PVT, Psychomotor Vigilance Test; RCT, randomised controlled trial.

## AUTHOR CONTRIBUTIONS

Damian M. Bailey and Adnan Haq conceived the idea. Adnan Haq and Damian M. Bailey wrote the first draft of the manuscript and versions thereof. Both authors approved the final version submitted for publication and agree to be accountable for all aspects of the work in ensuring that questions related to the accuracy or integrity of any part of the work are appropriately investigated and resolved. All persons designated as authors qualify for authorship, and all those who qualify for authorship are listed.

## CONFLICT OF INTEREST

D.M.B. is Editor‐in‐Chief of *Experimental Physiology*, Chair of the Life Sciences Working Group, member of the Human Spaceflight and Exploration Science Advisory Committee to the European Space Agency, member of the Space Exploration Advisory Committee to the UK and Swedish National Space Agencies and member of the National Cardiovascular Network for Wales and South‐East Wales Vascular Network.

## FUNDING INFORMATION

D.M.B. is supported by a Royal Society Wolfson Research Fellowship (Grant No. WM170007).
